# Dysbiosis of the gut microbiome is associated with CKD5 and correlated with clinical indices of the disease: a case–controlled study

**DOI:** 10.1186/s12967-019-1969-1

**Published:** 2019-07-17

**Authors:** Yang Li, Xinhuan Su, Lei Zhang, Yanwei Liu, Min Shi, Chenxiao Lv, Ying Gao, Dongmei Xu, Zunsong Wang

**Affiliations:** 1 Department of Nephrology, Shandong Provincial Qianfoshan Hospital, The First Hospital Affiliated with Shandong First Medical University, No. 16766 Jingshi Road, Jinan, 250014 Shandong China; 2 Department of Endocrinology, Shandong Provincial Hospital Affiliated to Shandong University, Shandong Provincial Key Laboratory of Endocrinology and Lipid Metabolism, Institute of Endocrinology and Metabolism, Shandong Academy of Clinical Medicine, Jinan, Shandong China; 3 Beijing Advanced Innovation Center for Big Data-Based Precision Medicine, School of Chemistry and Environment, Beihang University, Beijing, 100191 China; 4 Shandong Children’s Microbiome Center, Qilu Children’s Hospital of Shandong University, Jinan, 250022 China; 5 Department of Nephrology, Feicheng Mining Center Hospital, Feicheng High-Tech Development Zone, Taian, 271600 Shandong China; 6 Jinan Center for Food and Drug Control, Jinan, 250102 China; 7 Weifang Medical University, No. 7166 Baotong West Street, Weifang, 261053 Shandong China

**Keywords:** Gut microbiome, Chronic kidney disease, 16S rRNA gene sequencing, Indoxyl sulfate, p-Cresyl sulfate

## Abstract

**Background:**

Chronic kidney disease (CKD) is a universal chronic disease in China. The balance of the gut microbiome is highly crucial for a healthy human body, especially for the immune system. However, the relationship between the gut microbiome and CKD has not yet been clarified.

**Methods:**

A total of 122 patients were recruited for this study. Among them, 24 patients were diagnosed with CKD5 but did not receive hemodialysis therapy, 29 patients were diagnosed with CKD5 and received hemodialysis therapy and 69 were matched healthy controls. The gut microbiome composition was analyzed by a 16S rRNA (16S ribosomal RNA) gene-based sequencing protocol. High-performance liquid chromatography–electrospray ionization-tandem mass spectrometry (HPLC/ESI-MS/MS) technology was used to evaluate the levels of microbiome-related protein-binding uremic toxins level, indoxyl sulfate (IS) and p-cresyl sulfate (PCS), in the patients.

**Results:**

We compared the gut microbiome results of 122 subjects and established a correlation between the gut microbiome and IS and PCS levels. The results indicated that alpha and beta diversity were different in patients with CKD5 than in the healthy controls (*p *< 0.01). In comparison to healthy controls, CKD5 patients exhibited a significantly higher relative abundance of Neisseria (*p* < 0.001), Lachnoclostridium (*p* < 0.001) and Bifidobacterium (*p *< 0.001). Faecalibacterium (*p *< 0.001) displayed a notably lower relative abundance for CKD5 patients both with and without hemodialysis than for controls. It was also found that the concentrations of IS and PCS were correlated with the gut microbiome.

**Conclusions:**

Our results indicate that CKD5 patients both with and without hemodialysis had dysbiosis of the gut microbiome and that this dysbiosis was associated with an accumulation of IS and PCS. These results may support further clinical diagnosis to a great extent and help in developing potential probiotics to facilitate the treatment of CKD5.

**Electronic supplementary material:**

The online version of this article (10.1186/s12967-019-1969-1) contains supplementary material, which is available to authorized users.

## Background

With technological advancement, the relationship between the composition of the human microbiome, including the gut microbiome and corresponding diseases has been explored gradually and studied extensively [1]. The intestinal tract, which is the biggest digestive organ in a human body, harbors trillions of bacteria whose genomes contain 100 times more gens than that of humans [2]. Human intestinal tract fosters a number of phyla including Firmicutes, Bacteroidetes, Actinobacteria and Proteobacteria [3]. Recently, it was reported that variation in the gut microbiome was connected with chronic kidney disease (CKD) [4]. Chronic kidney disease has become a public health issue of global concern due to its increasing prevalence, high treatment cost and numerous complications. According to statistical analyses, the prevalence of CKD is 10.8% in China and 8–16% worldwide, and this number is growing [5, 6]. A study covering Europe, America and Asia has shown that the prevalence of CKD in elderly people over 64 years old is 23.4% to 35.8% [7]. As the disease progresses, kidney function is gradually lost, and eventually, the patient can receive only dialysis or kidney transplantation. Regardless of either of the treatment plans, the cost is quite large, and the quality of life of patients is greatly affected. Meanwhile, cardiovascular complications are one of the most important causes of death in patients with chronic kidney disease. In patients with stage 5 chronic kidney disease, there are many harmful substances known as uremic toxins, which are one of the main causes of cardiovascular complications. It is well known that protein-binding uremic toxin production increases in patients with CKD5, of which the most representative examples are IS and PCS. These substances have been proven to promote renal fibrosis, the risk of cardiovascular disease, insulin resistance and chronic kidney disease mineral and bone disorder (CKD-MBD) [8–10], and they could be influenced by the composition of the gut microbiome [11]. At the same time, the gut microbiome could affect the health of the kidney and thus even the whole body by the kidney-gut axis [12]. Some studies of changes in the gut microbiome in CKD patients have been reported [13], but the exact relationship between the gut microbiome and CKD5, especially in Chinese people, has not been validated clearly.

Therefore, in this study, we analyzed the gut microbiome of CKD5 (with and without hemodialysis) patients and healthy controls by sequencing the 16S ribosomal RNA (rRNA) gene. The results revealed effective biomarkers of the disease while revealing significant information about variation in the gut microbiome based on structure, composition and function. Some specific microbial biomarkers of CKD5 were evaluated and correlated with certain clinical indices.

## Methods

### Study subjects

Following approval by the Medical Ethics Committee of Shandong Provincial Qianfoshan Hospital (2017-S-015), we registered on the Chinese Clinical Trial Registry (ChiCTR1800015221) and recruited 122 individuals at Shandong Provincial Qianfoshan Hospital based on the diagnostic criteria for CKD in adults outlined in the KDIGO [14]. Among these individuals, 24 patients suffered from CKD5 but did not receive hemodialysis (CKD5-NHD group, C), 29 patients were diagnosed with CKD5 and had received regular dialysis therapy for more than 3 years (three times per week, CKD5-HD group, H), and 69 were healthy controls (normal group, N). All patients received regular phosphorus binder treatment, and all individuals resided in local areas and had similar eating habits. All individuals were fully informed of the experimental content and signed an informed consent form. Basic information from the clinical registration included the following: (1) general information: name, age, gender, contact information and place of birth; (2) history of medication: use of antibiotics, prebiotics/probiotics, medicinal activated carbon and other similar drugs within 1 month; and (3) clinical information: estimated glomerular filtration rate (eGFR) (according to the CKD-EPI formulation), serum creatinine (SCr), blood urea nitrogen (BUN), the presence of constipation, diarrhea or other types of intestinal diseases, family genetic disease or other diseases and infectious diseases, such as viral hepatitis.

The exclusion criteria included the following: (1) long-term constipation, chronic diarrhea or other types of intestinal diseases; (2) antibiotic, probiotic, prebiotic or synbiotic use 4 weeks before sample collection; (3) collection of specimens, including but not limited to polyethylene glycol electrolyte dispersants, enemas and other laxative drugs; (4) family hereditary kidney disease; and (5) viral hepatitis or other infectious diseases. The basic information for the patients and healthy controls is shown in Table 1.

**Table 1:** Statistics of the basic data collected from the patients and healthy controls

	CKD5-NHD group (n = 24)	CKD5-HD group (n = 29)	Normal group (n = 69)	*p* values
Gender (male/female)	12/12	17/12	27/42	0.190
Age (year)	55.50 (48.25–64.50)	54.00 (41.50–69.00)	51.00 (39.50–64.00)	0.367
EGFR (ml/min/1.73 m^2^)	5.33 (4.31–7.72)	5.75 (4.35–8.26)	126.07 (102.80–148.65)	0.000
SCr (μmol/l)	781.35 (595.58–921.75)	704.60 (563.10–899.95)	57.00 (48.15–66.00)	0.000
BUN (μmol/l)	23.95 (19.13–29.60)	23.50 (16.20–32.15)	4.90 (3.85–6.10)	0.000

### Biospecimen collection, DNA extraction and sequencing

Fecal specimens were collected in sterilized 2-ml tubes containing pure ethanol on ice, immediately frozen (within 30 min) and stored at − 80 °C until analysis. Genomic DNA was extracted using the cetyl trimethylammonium bromide (CTAB) method [15]. A NanoDrop 2000 (Thermo Electron Corporation, USA) spectrophotometer was used to determine the concentration of the extracted DNA. The V1–V2 regions of the 16S rRNA gene were amplified and sequenced on an Illumina HiSeq 2500 system. According to the reported bacterial 16S rRNA gene sequences in GenBank, we used DNAMAN V6 (Chinese version) software (Lynnon Biosoft Company, USA) to compare the homology of the bacteria and chose the highly conserved V1–V2 region to design a pair of primers (F: 5′-AGAGTTTGATCMTGGCTCAG-3′ and R: 5′-GCTGCCTCCCGTAGGAGT-3′) according to the basic principles of primer design [16]. PCR amplification was performed on the target fragments. Follow-up experiments were carried out according to the sequencing manual.

## 16S rRNA gene sequence analysis

The 16S sequencing paired-end data set was joined and quality filtered using the Laser FLASH method [17]. All sequences were analyzed using the Quantitative Insights into Microbial Ecology (QIIME, version 1.9.1) software suite [18]. Sequences were clustered against the Greengenes (13.8 release) ribosomal database’s 97% reference data set (http://greengenes.secondgenome.com/downloads). Sequences that remained unmatched with any of the entries in this reference were subsequently clustered into de novo OTUs at 97% similarity with the UCLUST algorithm. Taxonomy was assigned to all OTUs using the RDP classifier [19] within QIIME and the Greengenes reference data set. Rarefaction and rank abundance curves were calculated from OTU tables using alpha diversity and rank abundance scripts within the QIIME pipeline. The hierarchical clustering based on population profiles of the most common and abundant taxa was performed using UPGMA (unweighted pair group method with arithmetic mean, also known as average linkage) clustering on the distance matrix of OTU abundance. This method resulted in a Newick-formatted tree, which was obtained utilizing the QIIME package. Furthermore, Calypso online software (version 8.20, http://cgenome.net/wiki/index.php/Calypso) and R software (version 3.5.1) were used to analyze alpha diversity (Shannon, ACE, Chao1), beta diversity [unweighted/weighted UniFrac, principal coordinate analysis (PCoA)] and correlations between biomarkers and clinical indices. Linear discriminant analysis (LDA) and effect size (LEfSe) were performed with the online software Galaxy (http://huttenhower.sph.harvard.edu/galaxy/).

### IS and PCS determination and analysis

In the morning, approximately 4 ml of fasting (for at least 8 h before the blood collection) blood per individual was collected into a vacuum blood collection tube. The collected blood samples were allowed to stand for 30 min and then centrifuged at 1300×*g* for 10 min at room temperature using a conventional centrifuge. The supernatant (serum) of the sample after centrifugation was placed in a 1.5 ml cryotube. Then, 50.0 mg of an IS standard (purity: 99.8%, APExBIO Company, USA) was accurately weighed and diluted with methanol in a 50 ml volumetric flask (1 mg/ml). Next, 1.0 ml of the above solution was accurately pipetted, placed in a 10 ml volumetric flask, and diluted to the mark (100 μg/ml) with methanol. The solution was then diluted with physiological saline to an IS standard solution of 0.5, 1, 2, 10, 20, and 50 μg/ml. After that, 10.0 mg of hydrochlorothiazide standard (purity: 99.5%, APExBIO Company) was accurately weighed, diluted with methanol in a volumetric flask of 100 ml, and diluted to 0.05 μg/ml with methanol. The same method was used to obtain a PCS standard solution, and the internal standard for PCS was warfarin (purity: 99.2%, APExBIO Company) rather than hydrochlorothiazide. All solutions were stored at 4 °C in the dark. The participants’ sera were accurately absorbed in 100 µl and placed in a 1.5 ml centrifuge tube, the 500 µl internal standard (0.05 µg/ml hydrochlorothiazide or warfarin methanol solution) was precisely added and then vortexed for 1 min and centrifuged at 4500×*g* for 10 min at 4 °C. Next, 100 µl of the supernatant was mixed with an equal volume of water, placed in an auto injection bottle, and injected 5 µl, and the IS content in human serum was calculate by the internal standard method. HPLC/ESI-MS/MS conditions were as follows. (1) Chromatographic conditions: Shimadzu Shim-pack XR-ODS (75 mm * 2.0 mm, 2.1 μm); mobile phase A (0.1% aqueous formic acid, containing 2 mM ammonium acetate): B (gradient elution with acetonitrile); flow rate of 0.3 ml/min; column temperature of 40 °C; and injection volume of 5 μl. (2) Mass spectrometry conditions: the ion source was an electrospray ionization (ESI) source; the detection method was negative ion detection; the scanning mode was multireaction monitoring (MRM); the injection voltage was − 4500 V; the ion source temperature was 550 °C; the air curtain gas flow rate was 30 Psi; the atomizing gas flow rate was 55 Psi; the collision gas flow rate was 8 Psi; and the scanning time was 0.2 s. The ion conditions for quantification and characterization are shown in Tables 2 and 3.

**Table 2:** IS and internal standard mass spectrometry ion conditions

	Q1 (m/z)	Q3 (m/z)	CE	DP	EP	CXP
IS	212.6	79.9	− 55	− 50	− 10	− 11
Internal standard	295.8	268.9	− 25	− 100	− 10	− 11

*CE* collision energy, *DP* cluster voltage, *EP* Q0 voltage, *CXP* impact chamber outlet voltage

**Table 3:** PCS and internal standard mass spectrometry ion conditions

	Q1 (m/z)	Q3 (m/z)	CE	DP	EP	CXP
PCS	186.8	107.0	− 26	− 40	− 10	− 11
Internal standard	307.0	249.8	− 35	− 40	− 10	− 11

*CE* collision energy, *DP* cluster voltage, *EP* Q0 voltage, *CXP* impact chamber outlet voltage

The IS and PCS standard solutions of 0.5, 1, 2, 10, 20, and 50 μg/ml were used separately and processed according to the abovementioned operation steps for HPLC analysis. A standard curve (weighting coefficient was 1/C) was fitted by a linear regression method with the ratio of IS (PCS) to the internal standard peak area (Y) versus IS (PCS) concentration (C, μg/ml).

### Statistical analysis

The clinical characteristics of the subjects are represented as the median (quartile interval) [M(IQR)] and the relative numbers, depending on the data and distribution types. Depending on the characteristics of the measurement data, one-way ANOVA was applied to multiple groups. The data from multiple groups of classified data were compared with the R*C Chi square test. Diversity categorization of alpha and beta diversity was defined in the OTU table to a sequencing depth of 20,000 per sample. Moreover, alpha diversity was determined using the Mann–Whitney U test, and beta diversity was acquired by ANOSIM (analysis of similarities). LEfSe combines the Kruskal–Wallis test or pairwise Wilcoxon rank sum test with linear discriminant analysis (LDA), whose threshold value on the logarithmic LDA score equals 3.0. Spearman’s rank correlation method was used to analyze the relationship between the microbiome and clinical indices. Analyses were performed using the SPSS statistical package (version 17.0), R software (version 3.5.1), Galaxy online software, Calypso online software (version 8.20) and GraphPad Prism 7 software. *p* values less than 0.05 were considered statistically significant.

## Results

To establish the gut microbiome characteristics of patients with CKD5, we conducted 16S rDNA gene sequencing to analyze 122 fecal samples from 122 individuals (24 CKD5 patients without dialysis, 29 CKD5 patients with dialysis and 69 healthy controls). The clinical characteristics of the subjects are summarized in Table 1. No significant differences were noticed in either age or gender among the three groups. Through preprocessing of the sequencing data, 6,577,239 high-quality sequences (Phred ≥ Q30) with an average of 548,103 sequences per sample were obtained.

### Gut microbiome composition of the CKD5-NHD, CKD5-HD and healthy control subjects

**Fig. 1:**
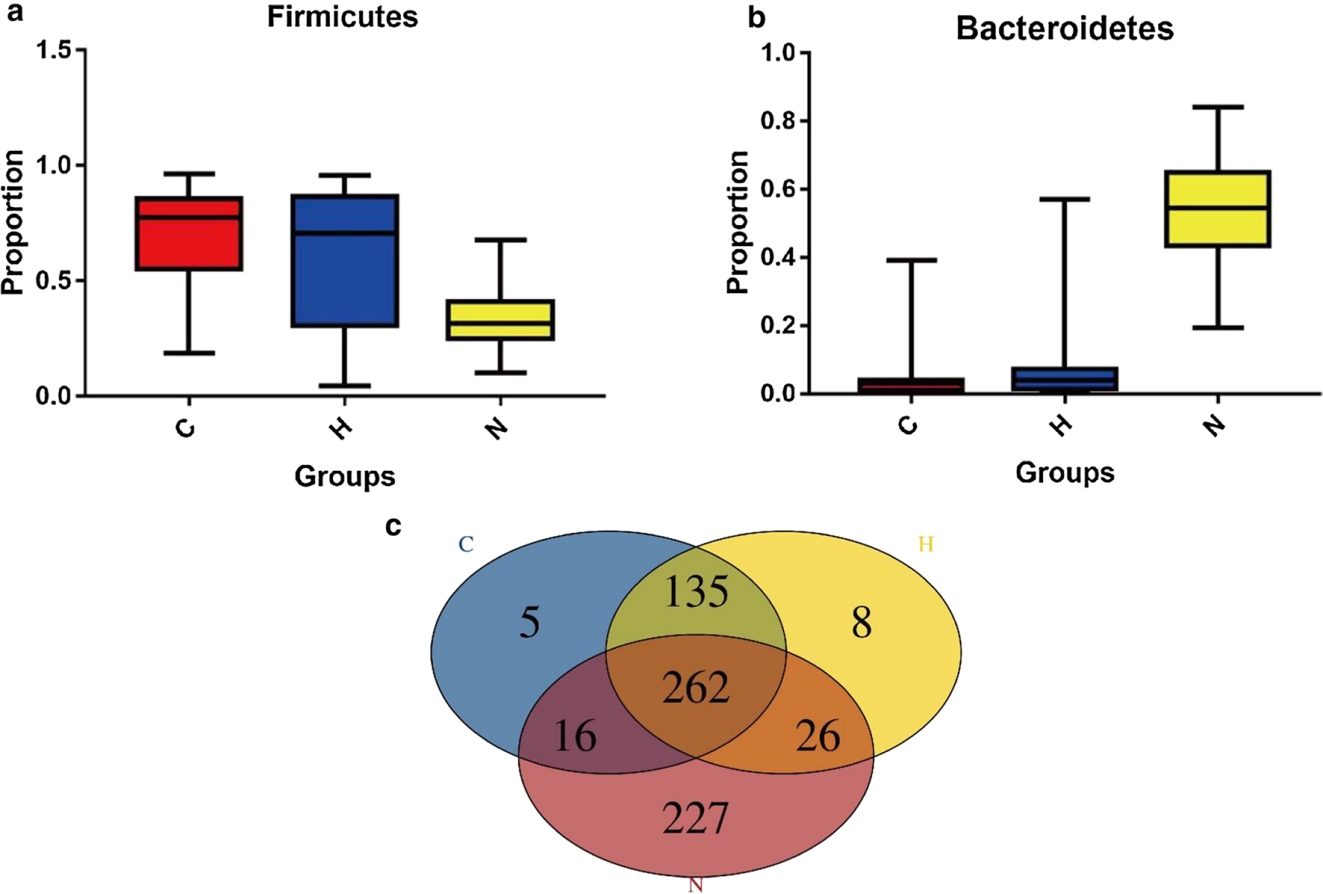
Comparison of relative taxon abundances of the phyla Firmicutes and Bacteroidetes among the CKD5-NHD, CKD5-HD, and healthy control subjects. **a** Comparison of relative abundances of the phylum Firmicutes among the CKD5-NHD, CKD5-HD, and healthy control subjects. **b** Comparison of relative abundances of the phylum Bacteroidetes among the CKD5-NHD, CKD5-HD, and healthy control subjects. **c** Venn diagram. *C* CKD5-NHD group, *H* CKD5-HD group, *N* normal group

To characterize the community attributes of the gut microbiome of individuals with CKD5, relative taxon abundances of the phyla Firmicutes and Bacteroidetes of the CKD5-NHD, CKD5-HD and healthy control groups were compared (Fig. 1a, b). Venn diagrams distinguished between the gut microbiomes of the three groups (Fig. 1c). Within the healthy control group, 227 unique species were found. Similarly, 5 unique species were found in the CKD5-NHD group, while 8 unique species were found in the CKD5-HD group (details shown in Additional file 1).

### Variations in the gut microbiome among the three groups

**Fig. 2:**
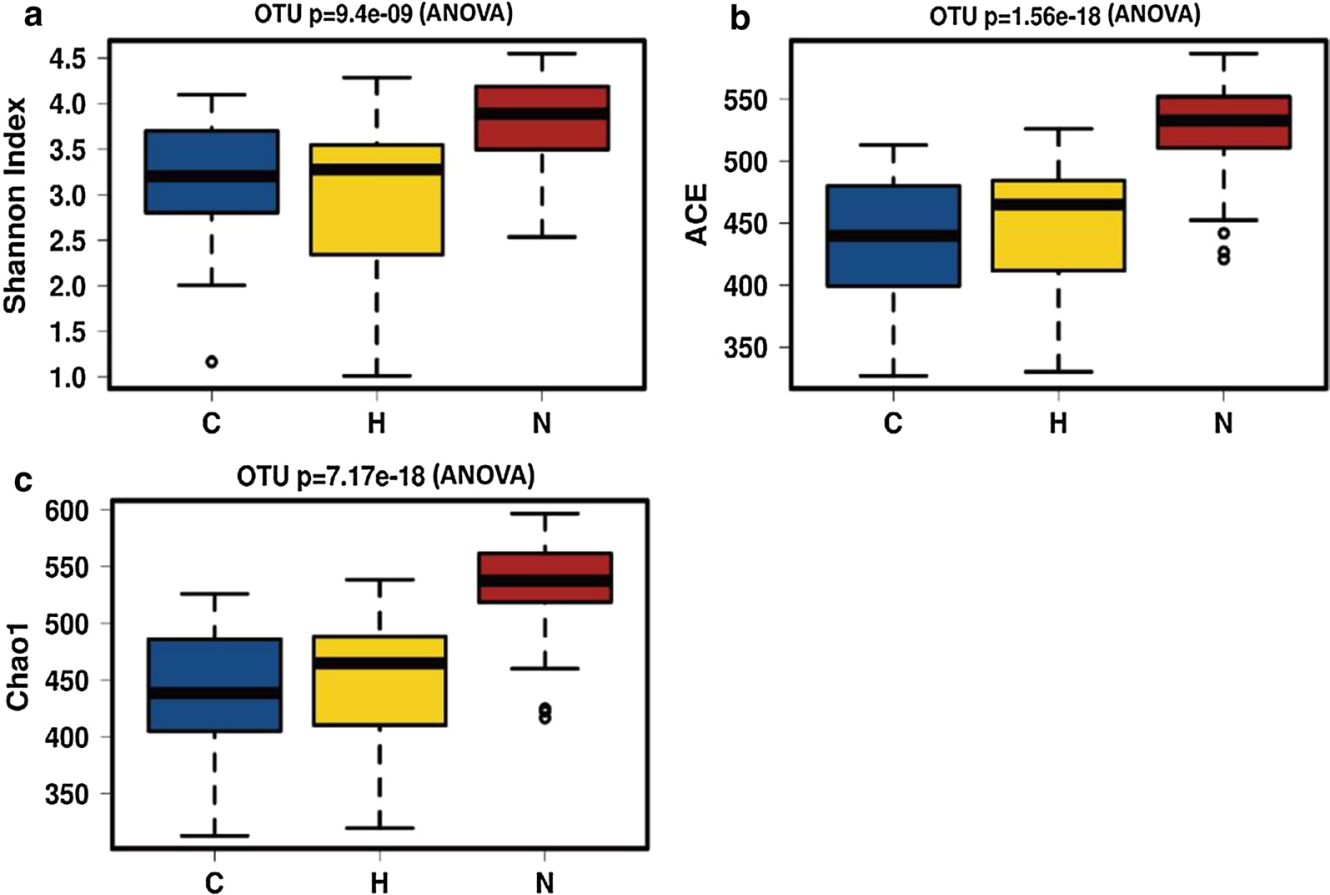
Phylogenetic diversity of the gut microbiome among the CKD5-NHD, CKD5-HD and healthy control subjects. Box plots depict microbiome diversity and abundance differences according to the Shannon (**a**), ACE (**b**) and Chao1 (**c**) indices among the CKD5-NHD, CKD5-HD and healthy control subjects. *C* CKD5-NHD group, *H* CKD5-HD group, *N* normal group

**Fig. 3:**
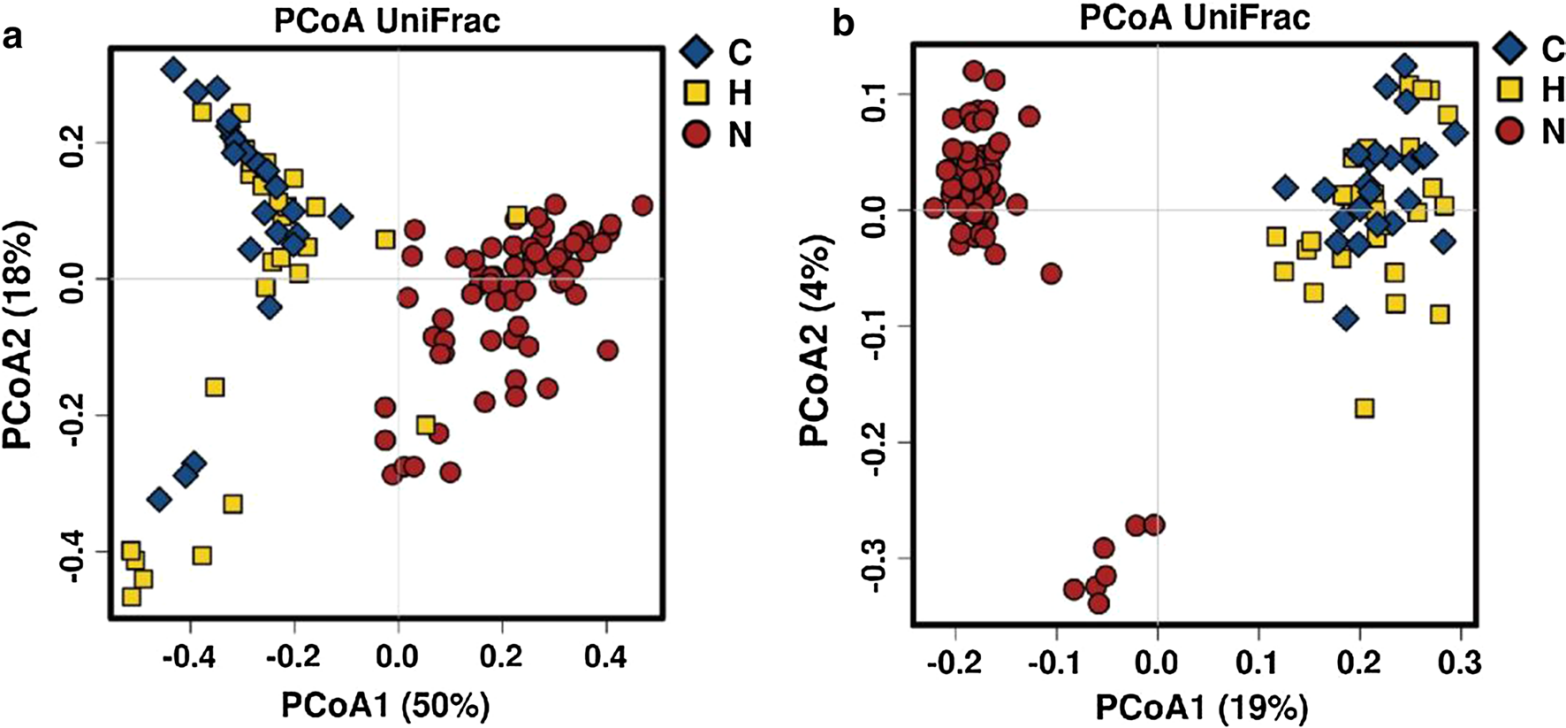
PCoA of the microbiome among the CKD5-NHD, CKD5-HD and healthy control subjects. **a** Weighted UniFrac PCoA. **b** Unweighted UniFrac PCoA. *C* CKD5-NHD group, *H* CKD5-HD group, *N* normal group

We analyzed the alpha and beta diversity to explore the variations in the gut microbiome among the three groups. Significant differences were found among the healthy control, CKD5-NHD and CKD5-HD groups with respect to microbial diversity (Shannon index, *p *< 0.001) and microbial abundance (ACE index, *p *< 0.001; Chao1 index, *p *< 0.001). Microbial diversity and abundance were higher in the healthy control group than in both CKD5 groups (Fig. 2). In addition, we calculated the unweighted and weighted UniFrac distances to investigate the correlation of the gut microbiome between the CKD5 groups and the healthy control group. These calculations revealed that the healthy control group was significantly different from the CKD5-NHD group and CKD5-HD group (Fig. 3). Together, the results indicated a significant variation in the gut microbiome between the CKD5 disease and healthy control groups.

### Bacterial taxonomic differences among the three groups

**Fig. 4:**
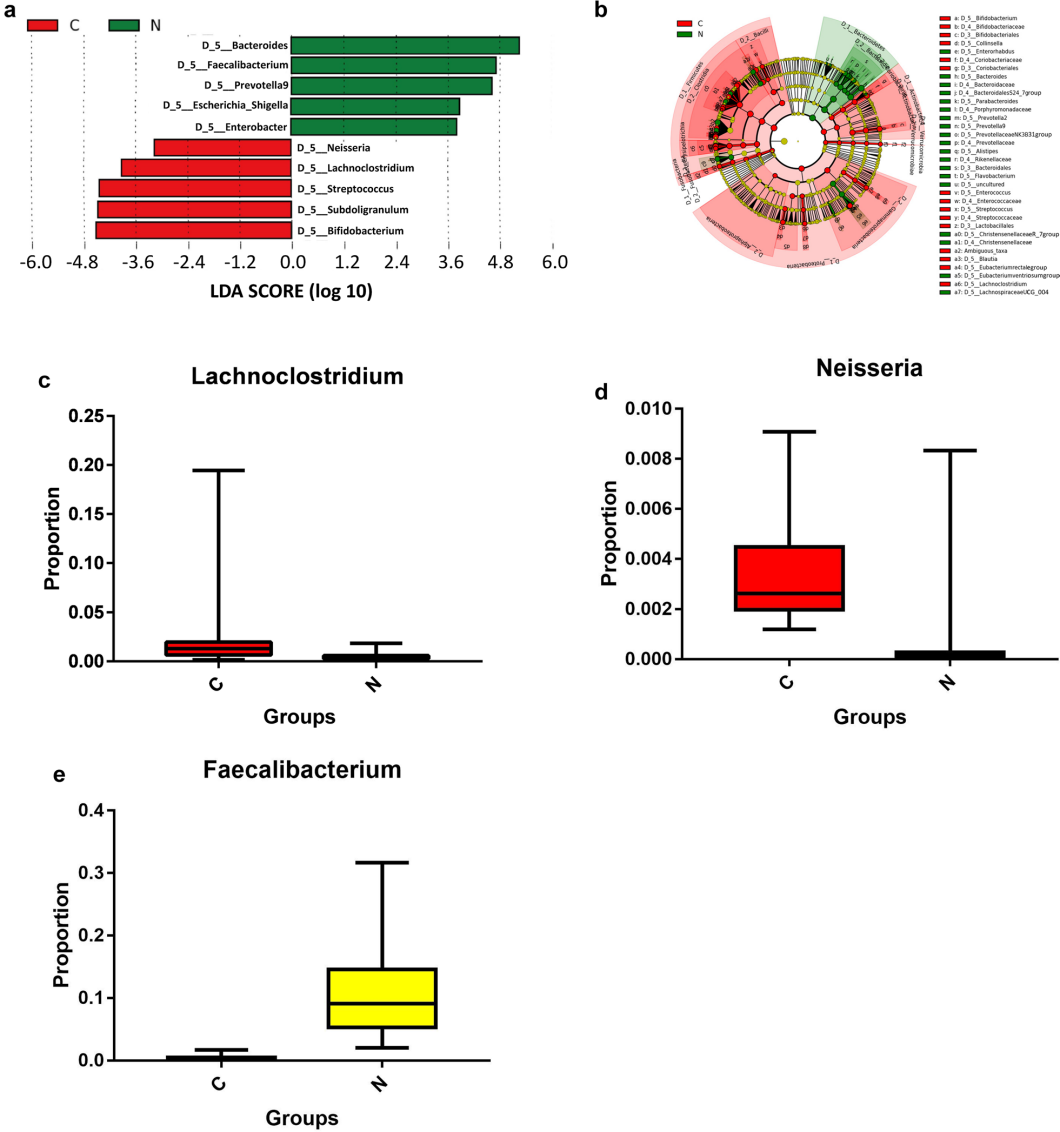
Characteristics of the microbial community composition in the CKD5-NHD and healthy control groups. **a** The major differentially abundant taxa between CKD5-NHD and healthy control subjects (LDA score above 3.0), which was generated from LEfSe analysis. **b** The enriched taxa in the CKD5-NHD and healthy control gut microbiomes are represented in the cladogram. The central point represents the root of the tree (bacteria) and each ring represents the next lower taxonomic level (order to genus: D_3 order, D_4 family, D_5 genus). The diameter of each circle represents the relative abundance of the taxon. **c**–**e** Relative abundance of taxa between the CKD5-NHD and healthy control subjects was compared, and all the *p* values were less than 0.0001 (Mann–Whitney U test). *C* CKD5-NHD group, *N* normal group

**Fig. 5:**
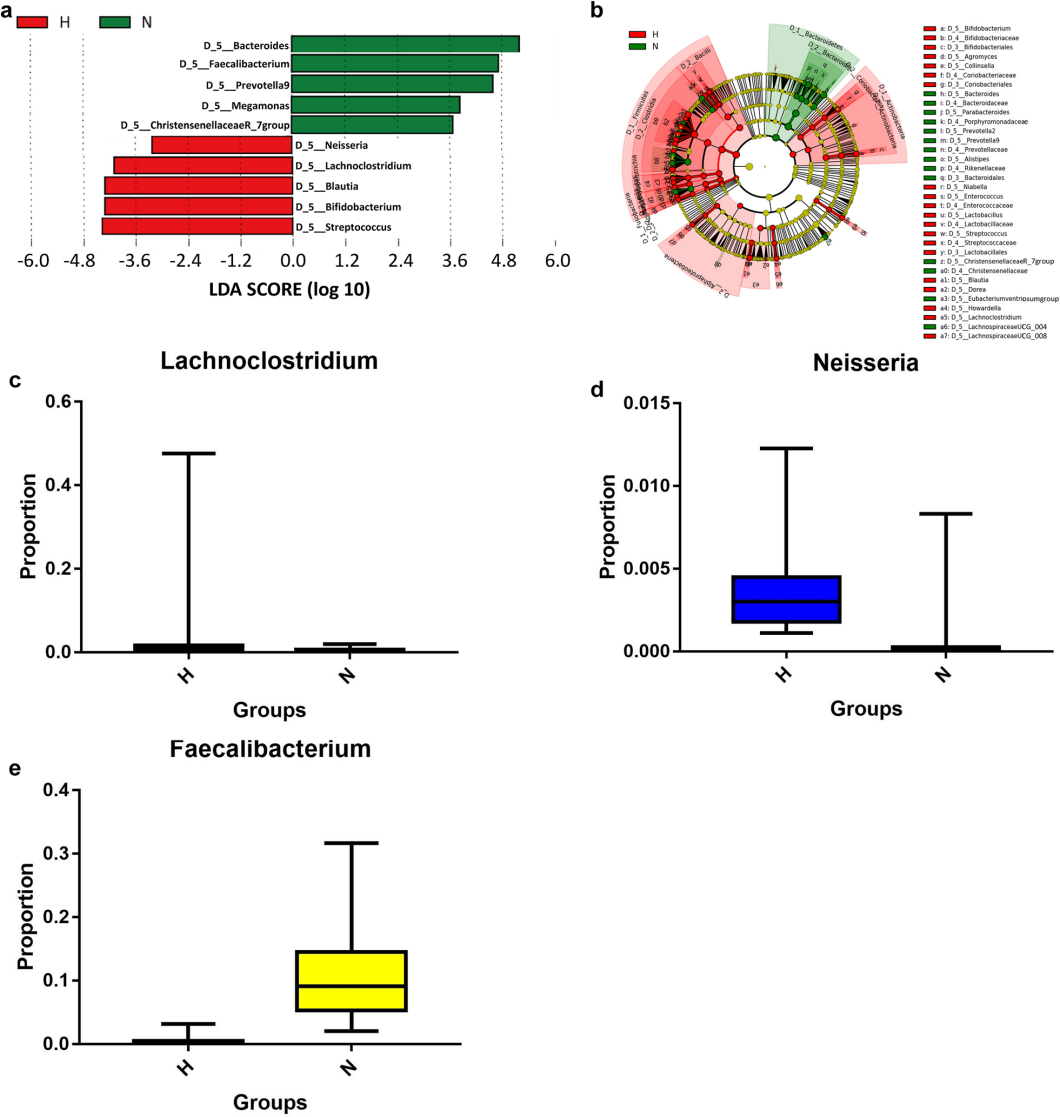
Characteristics of the microbial community composition in the CKD5-HD and healthy control groups. **a** The major differentially abundant taxa between CKD5-HD and healthy control subjects (LDA score above 3.0), which was generated from LEfSe analysis. **b** The enriched taxa in the CKD5-HD and healthy control gut microbiomes are represented in the cladogram. The central point represents the root of the tree (bacteria), and each ring represents the next lower taxonomic level (order to genus: D_3 order, D_4 family, D_5 genus). The diameter of each circle represents the relative abundance of the taxon. **c**–**e** Relative abundance of taxa between the CKD5-HD and healthy control subjects was compared (Mann–Whitney U test). *H* CKD5-HD group, *N* normal group

To select biomarkers from the disease groups, we used LEfSe to analyze and distinguish the composition of the gut microbiome between the CKD5 and healthy control groups. The gut microbiome of the CKD5-NHD group was characterized by a dominance of Lachnoclostridium, Neisseria, etc., whereas the microbiome in the healthy control group was dominated by the genus Faecalibacterium, etc. (p < 0.05, Fig. 4). The CKD5-HD group’s microbiome was also characterized by a dominance of Lachnoclostridium, Neisseria, etc., whereas the microbiome in the healthy control group displayed a dominance of Faecalibacterium, etc. (p < 0.05, Fig. 5). The images of Figs. 4a and 5a are two cut-off images from the integral LEfSe graph to show the major different taxa at the genus level. The integral graphs are shown in Additional files 2 and 3.

### The gut microbiome of CKD5 patients was associated with clinical indices

**Fig. 6:**
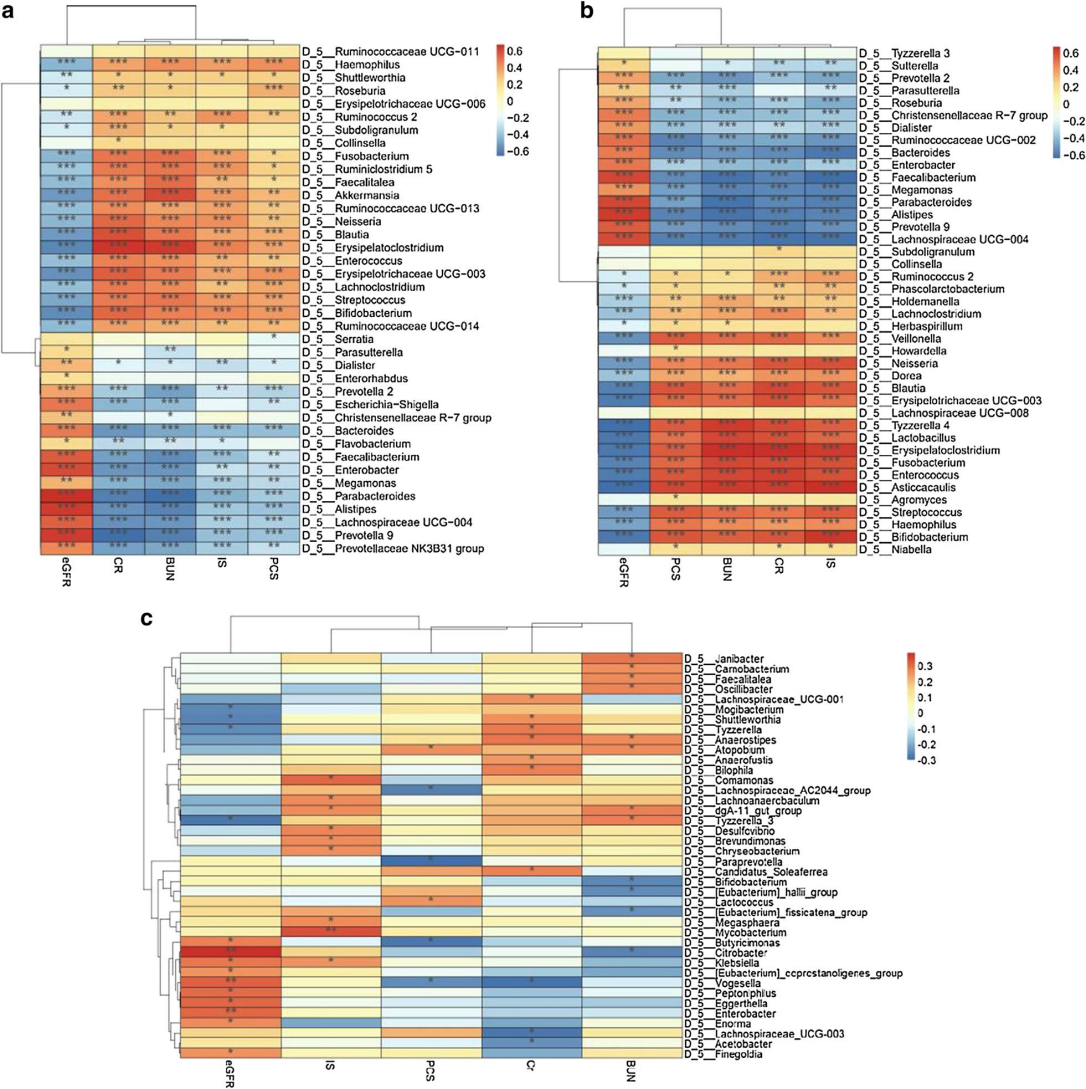
Heat map of Spearman’s correlation analysis between the gut microbiome of the three groups and the clinical indices. **a** Heat map of Spearman’s correlation analysis between the gut microbiome of CKD5-NHD subjects and the clinical indices. **b** Heat map of Spearman’s correlation analysis between the gut microbiome of CKD5-HD subjects and the clinical indices. **c** Heat map of Spearman’s correlation analysis between the gut microbiome of the normal group subjects and the clinical indices. *p < 0.05, **p < 0.01, ***p < 0.001

With HPLC/ESI-MS/MS technology, the IS and PCS concentrations and chromatogram (shown in Additional file 4) of serum samples in each group were obtained. The details of IS and PCS concentrations are shown in Table 4. The Spearman’s rank correlation coefficient method was used to evaluate the correlation between each subject’s gut microbiome and clinical index parameters, including eGFR, SCr, BUN, IS and PCS. The details are shown in Fig. 6. Strong correlations (correction r > 0.6 or r < − 0.6, p < 0.05) were found among 39 taxa and the 5 clinical indices in CKD5-NHD subjects (Fig. 6a). Similarly, a relationship between the biomarkers (41 taxa) and clinical indices (5 indices) in CKD5-HD subjects was established (Fig. 6b). IS, PCS, BUN and CR exhibited a significant positive correlation with the genera Enterococcus and Bifidobacterium, whereas eGFR showed a significant negative correlation with the two bacterial genera. However, no significant correlations (|r| < 0.4, p < 0.05) were found between the gut microbiome and clinical indices in the normal group. The details are described in the figure below.

**Table 4:** The IS and PCS concentrations in each group

	CKD5-NHD	CKD5-HD	Normal	*p* values
IS (μg/ml)	5.48 (1.10–12.55)	15.05 (10.45–22.37)	0.48 (0.33–0.90)	< 0.0001
PCS (μg/ml)	3.53 (70.8–10.74)	5.66 (2.77–10.89)	0.45 (0.19–0.77)	< 0.0001

## Discussion

In this study, an analysis of gut microbiome composition was performed for stool samples from subjects affected by CKD5 and matched healthy controls. It was found that the gut microbiome of patients with both CKD5-NHD and CKD5-HD demonstrated lower microbial richness as well as distinct composition compared to those of the healthy control group subjects. It was further revealed that the gut microbiome might be associated with certain enterogenous protein-binding uremic toxins. Our pilot study reported that the composition of the gut microbiome was correlated with CKD5 with and without hemodialysis.

Several studies have demonstrated that dysbiosis of human microbiome could be primarily caused by inflammatory disorders throughout the body [20–24]. In our study, we observed that the microbial richness was obviously lower in both the CKD5-NHD and CKD5-HD groups than in the healthy control group. A number of studies have suggested that greater overall diversity implies better health [25]. The assembled results indicate that an opportunistic pathogen may colonize patients suffering from CKD5 disease.

With respect to gut the microbiome composition, the relative abundance of Lachnoclostridium (*p* < 0.001) and Neisseria (*p* < 0.001) was significantly higher in both the CKD5-NHD and CKD5-HD groups than in the health control group. There were some differences between our results and the discovery of Vaziri et al. [13]. With regard to these differences, race, dietary habit, living environment and some other aspects may play critical roles. In our study, Neisseria levels were found to be elevated in the setting of esophageal inflammation, which was correlated with inflammatory disorders in pancreatic disease [26–29]. As we all know, patients with CKD are under the condition of a microinflammatory state. Neisseria may aggravate this pathological condition. Several studies also demonstrated that reduced bone volume of fat diet-induced bone loss in male mice was associated with the presence of Lachnoclostridium [30]. There were situations of dyslipidemia and mineral bone disorder (CKD-MBD) in patients with CKD5; Lachnoclostridium may be one of the culprits. Interestingly, Bifidobacterium, usually known as a kind of probiotic, was found to be significantly higher in the disease groups than in the healthy group. As we know, butyrate (BT) is one of the short chain fatty acids (SCFAs) that can maintain the health of the intestinal tract [31]. Adequate butyrate has been proven to be critical for sustaining the health of the gut microbiome. Butyrate not only maintains the integrity of the intestinal epithelium but also provides energy for respiration of intestinal epithelial cells and regulates immune responses of the intestinal tract [32, 33]. For instance, BT develops an antiinflammatory effect by inhibiting the recruitment and proinflammatory activity of immune cells, such as neutrophils. It could activate the differentiation of colonic Treg cells to suppress inflammation [34]. However, when the abundance of some bacteria, such as Bifidobacterium, increased, the abundance of bacteria that generate butyrate was significantly reduced which may damage epithelial cells and deteriorate the microinflammatory state. Vaziri et al. [35–37] found that ESRD patients exhibited significant expansion of bacterial families possessing urease, which resulted in the accumulation of some uremic toxins, such as IS and PCS, and the destruction of tight junctions. Alterations in the gut microbiome in both the CKD5-NHD and CKD5-HD groups may be due to intestinal microecological environmental changes. These changes may occur for several of the following reasons. ① The generally poor nutritional status of patients with injured renal function makes it difficult for a microbiome that is beneficial to the body to obtain sufficient nutritional substrates, which inhibits the growth of advantageous bacterial groups [38]. ② Because of restraint of the advantageous bacterial groups, intestinal peristalsis slows, intestinal microvilli are injured, and the intestinal clearance ability is reduced, which provides opportunities for pathogenic bacteria to contact and adhere to mucous membranes; this possibility is also an important reason for the changes in the microbiome structure [39]. ③ Renal dysfunction leads to the accumulation of waste material, and thus the intestinal mucosa becomes ischemic, the mucosal barrier is damaged, and the permeability of the mucous membrane increases; moreover, the growth environment for the bacteria changes due to underlying inflammation [40]. Our results showed an increase in these three genera of bacteria, which indicates that these bacteria could be a factor that affects renal function.

The gut microbiome plays a critical role in substance metabolism and influences the essential diagnosis and treatment of various pivotal diseases, such as cancer, diabetes and CKD. In this study, our results indicated that the gut microbiome was associated with the disease indices. IS and PCS were found to have a significant positive association with Enterococcus (*p* < 0.01) and Bifidobacterium (p < 0.001). It is known that IS and PCS are secreted and excreted primarily through the organic anion transporter (OAT) in renal tubules. The renal excretion function of CKD patients is impaired, resulting in a reduction in the excretion of the substance and its accumulation in the body [41]. Simultaneously, due to the structure and function of the microbiome disturbance, overgrowth of Enterococcus, Bifidobacterium and other phenolic- and indole-producing bacteria occurred in CKD patients, resulting in increased levels of IS, PCS and other harmful metabolites and further leading to the accumulation of these two substances. Studies have shown that IS and PCS promote renal fibrosis, myocardial apoptosis, and insulin resistance and affect osteoblast function [42–48]. BUN and CR are the two main indices that reflect kidney function and are filtrated by the glomerulus and partly excreted by the renal tubules. With renal (the decline of eGFR) and gut bacteria metabolism dysfunction, the concentrations of BUN and CR were increased. Due to the disordered internal environment, CKD patients usually have an intestinal microecological imbalance (i.e., proliferation of a large number of harmful bacteria and a reduction in beneficial bacteria), resulting in a state of long-term microinflammation within the body. Disordered microbiomes contribute to the state of CKD by causing the accumulation of IS, PCS, BUN, CR and other uremic toxins, thereby aggravating the illness and increasing the risk of a variety of complications, such as cardiovascular disease, and eventually increasing all-cause mortality. Therefore, modifying the gut microbiome by prebiotics may be a novel way to improve the disease state [49, 50].

## Conclusions

This study indicates that variation in the gut microbiome is associated with CKD5 disease. It also suggests the potential pathogens active in CKD. These results may be essential for clinical diagnosis and could be further used to develop potential probiotics that could facilitate the treatment of CKD. However, it remains unknown whether renal disorders cause dysbiosis or vice versa. Further studies need to scrutinize and explore the mechanisms between the gut microbiome and CKD using modeled organisms.

## Additional files

**Additional file 1.** The unique species in three groups.

**Additional file 2.** LEfSe graph of group C and N.

**Additional file 3.** LEfSe graph of group H and N.

**Additional file 4: Fig. S1.** IS chromatogram of the CKD5-HD group. **Fig. S2.** PCS chromatogram of the CKD5-HD group. **Fig. S3.** IS chromatogram of the CKD5-NHD group. **Fig. S4.** PCS chromatogram of the CKD5-NHD group.

## Data Availability

The datasets used and/or analyzed during the current study are available from the corresponding author on reasonable request.

## Abbreviations

CKD: chronic kidney disease
rRNA: ribosomal RNA
HPLC/ESI-MS/MS: high-performance liquid chromatography–electrospray ionization-tandem mass spectrometry
IS: indoxyl sulfate
PCS: p-Cresyl sulfate
CKD-MBD: chronic kidney disease mineral and bone disorder
eGFR: estimated glomerular filtration rate
SCr: serum creatinine
BUN: blood urea nitrogen
CTAB: cetyl trimethylammonium bromide
PCoA: principal coordinate analysis
LDA: linear discriminant analysis
LEfSe: linear discriminant analysis effect size
SCFAs: short chain fatty acids
OAT: organic anion transporter
