# Supplementary figures and images for: Dysbiosis of the gut microbiome is associated with CKD5 and correlated with clinical indices of the disease: a case–controlled study
Source: J Transl Med. 2019 Jul 17;17:228. doi: 10.1186/s12967-019-1969-1 (PMC6637476; doi:10.1186/s12967-019-1969-1)

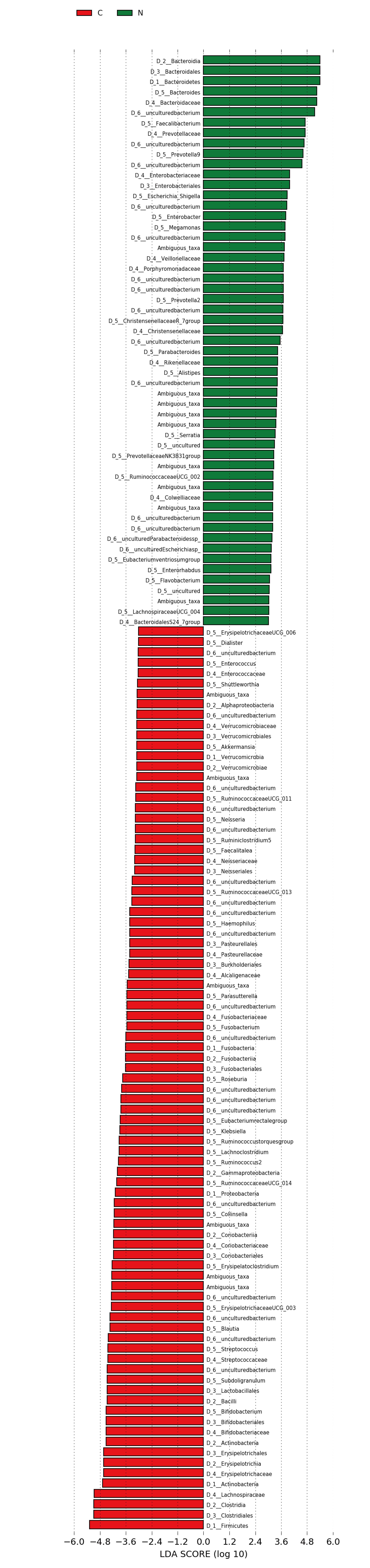

Supplement: Supplementary file 2 — Additional file 2. LEfSe graph of group C and N. [file 12967_2019_1969_MOESM2_ESM.jpg]

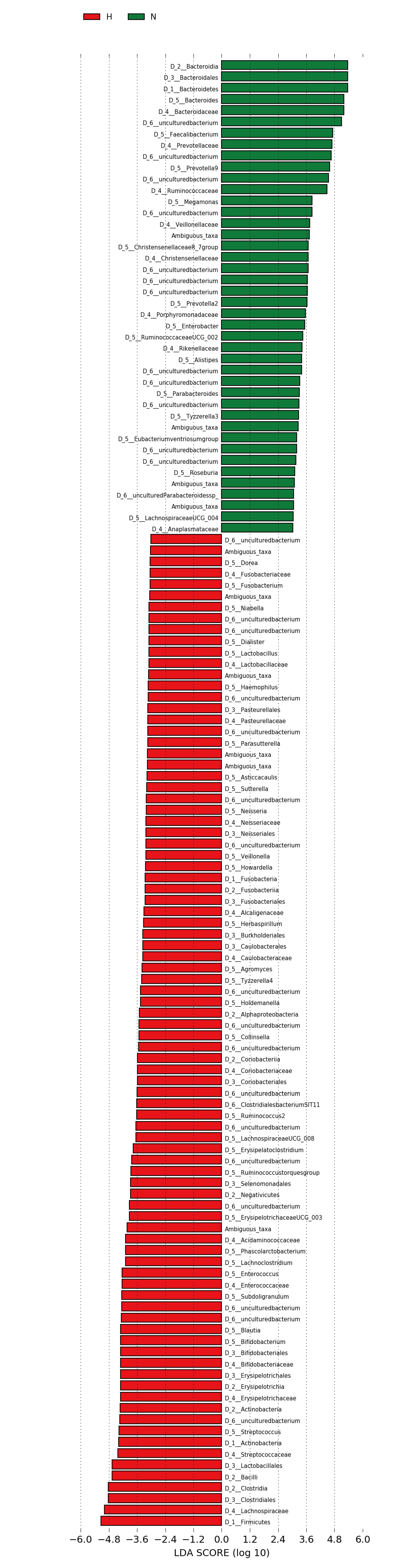

Supplement: Supplementary file 3 — Additional file 3. LEfSe graph of group H and N. [file 12967_2019_1969_MOESM3_ESM.jpg]
