# Supplementary material for: Dysbiosis of the gut microbiome is associated with CKD5 and correlated with clinical indices of the disease: a case–controlled study
Source: J Transl Med. 2019 Jul 17;17:228. doi: 10.1186/s12967-019-1969-1 (PMC6637476; doi:10.1186/s12967-019-1969-1)
Supplement: Supplementary file 4 — Additional file 4: Fig. S1. IS chromatogram of the CKD5-HD group. Fig. S2. PCS chromatogram of the CKD5-HD group. Fig. S3. IS chromatogram of the CKD5-NHD group. Fig. S4. PCS chromatogram of the CKD5-NHD group. [file 12967_2019_1969_MOESM4_ESM.doc]

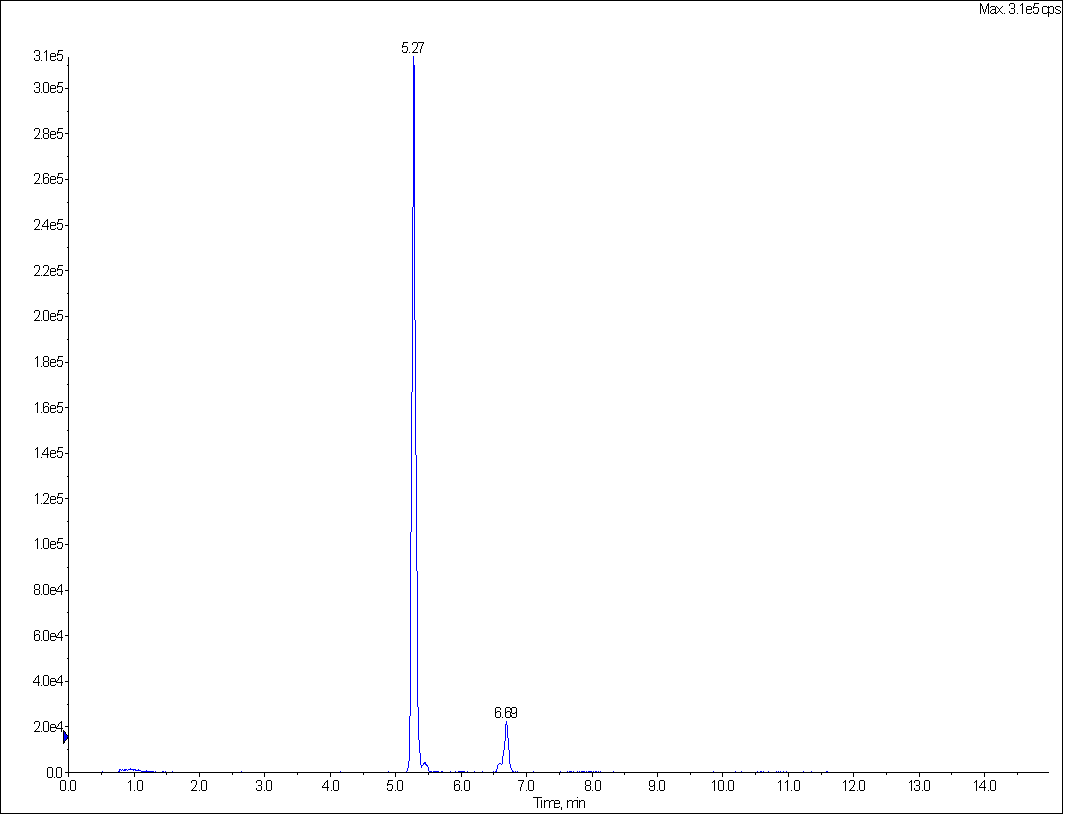


FIG S1. IS chromatogram of the CKD5-HD group


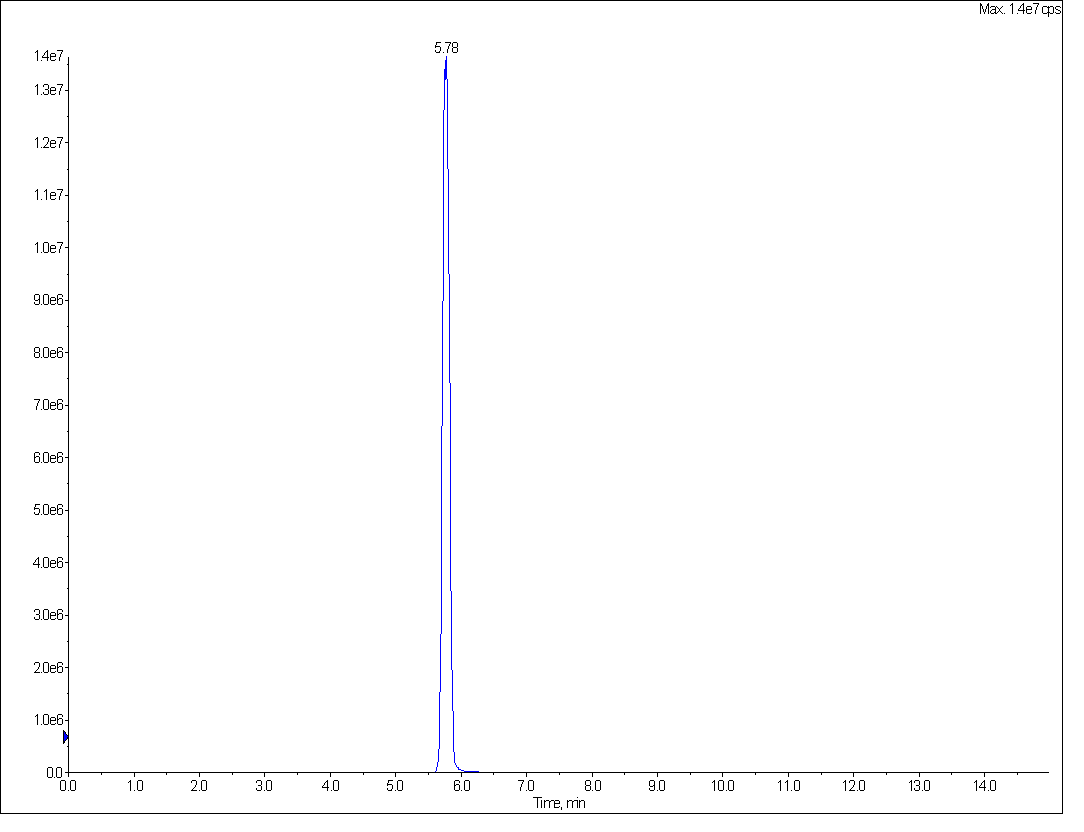


FIG S2. PCS chromatogram of the CKD5-HD group


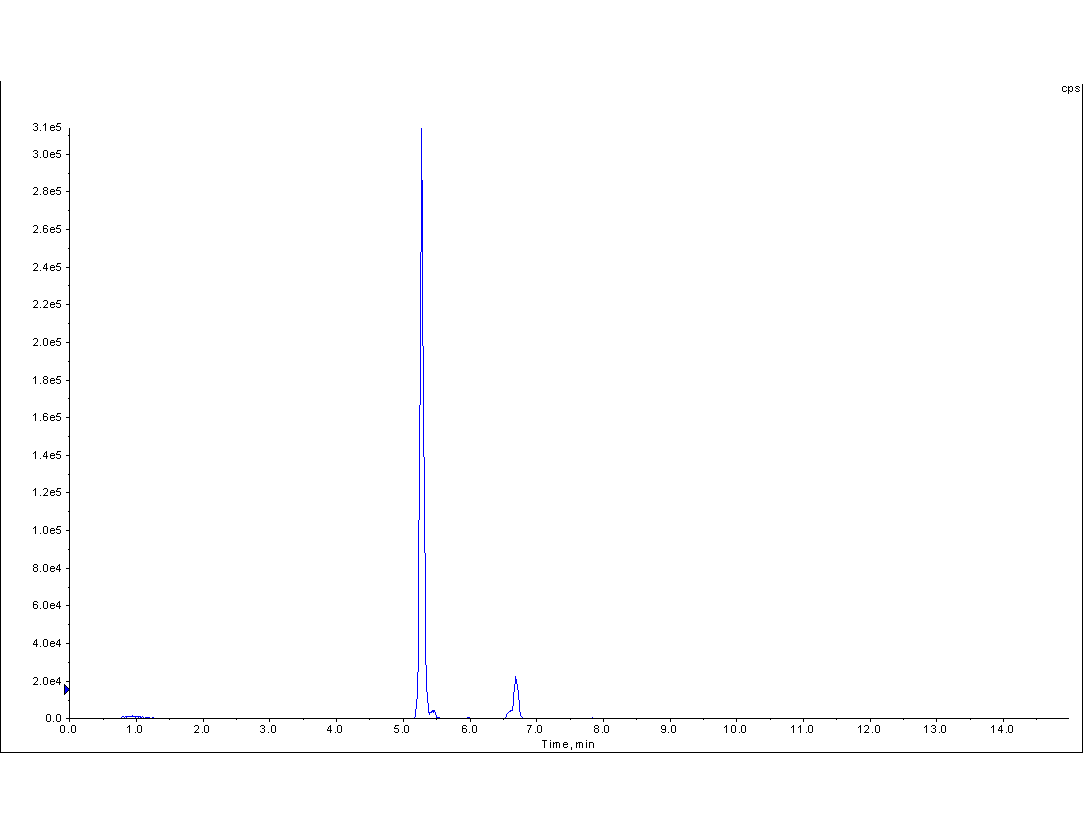


FIG S3. IS chromatogram of the CKD5-NHD group


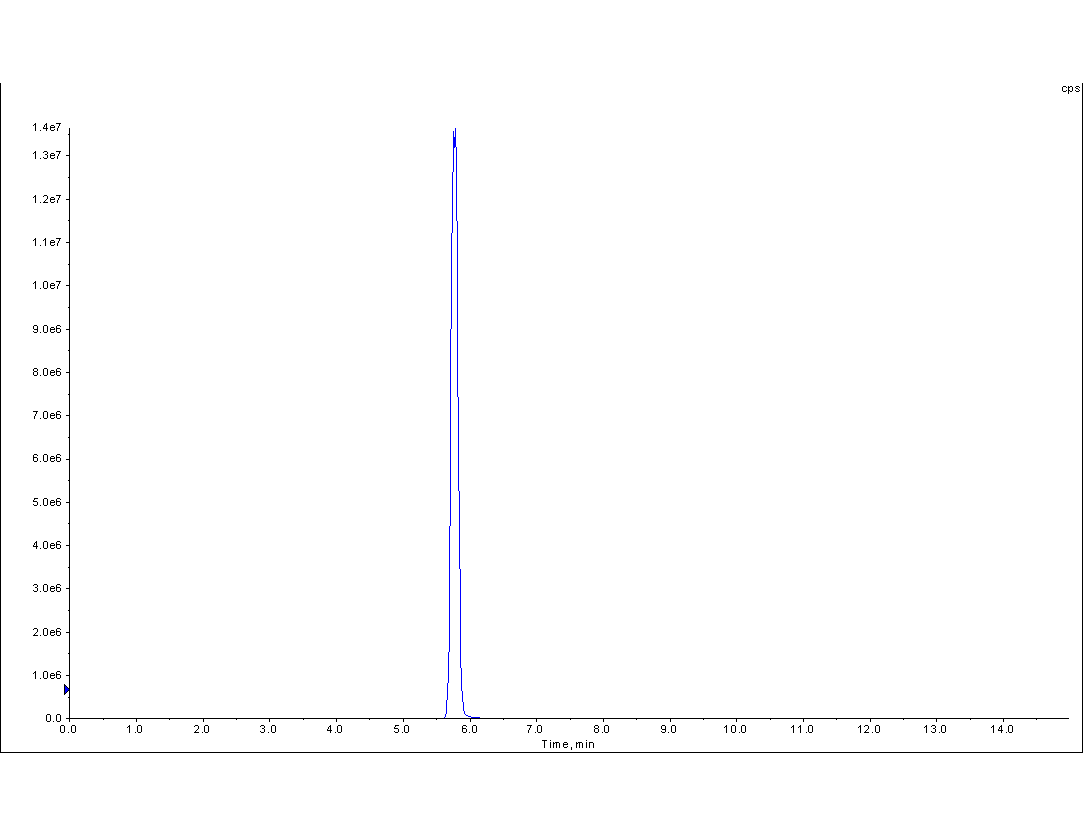


FIG S4. PCS chromatogram of the CKD5-NHD group
